# Comprehensive analysis of IGFL1 in colorectal cancer and its promotion of tumour progression via inhibition of lipophagy

**DOI:** 10.3389/fimmu.2025.1719525

**Published:** 2026-01-19

**Authors:** Ao Wang, Yu Zhou, Zhijuan Deng, Ying Duan, Zhenjie Dai, Dan Jiang

**Affiliations:** 1 Department of Pathology, West China Hospital, Sichuan University, Chengdu, Sichuan, China; 2 Department of Pathology, Affiliated Hospital of Panzhihua University, Panzhihua, Sichuan, China

**Keywords:** colorectal cancer, IGFL1, lipophagy, therapeutic target, tumor microenvironment

## Abstract

**Objective:**

Colorectal cancer (CRC) ranks among the most prevalent malignancies, with increasing incidence and mortality rates presenting a substantial public health challenge. While insulin growth factor like family member 1 (IGFL1) has been implicated in the regulation of various diseases, its functional role in colorectal cancer remains poorly characterised. This study therefore aims to elucidate the involvement of IGFL1 in CRC through an integrated approach combining bioinformatics analysis and experimental validation.

**Methods:**

The expression of IGFL1 in CRC and its association with clinicopathological features, diagnostic relevance, and patient prognosis were evaluated using data from The Cancer Genome Atlas (TCGA) and Gene Expression Omnibus (GEO) databases. Immunohistochemistry was performed to validate IGFL1 protein expression in CRC tissue samples. Immune cell infiltration levels and immune microenvironment scores related to IGFL1 expression were analysed using multiple computational algorithms, including CIBERSORT, ssGSEA, ESTIMATE, EPIC, MCP-counter, quanTIseq, TIMER, xCell, and CIBERSOR. Furthermore, IGFL1 expression patterns across distinct cellular subpopulations were examined using single-cell RNA sequencing datasets from the Tumor Immune Single-cell Hub (TISCH) database. The TIDE algorithm was applied to assess the potential clinical efficacy of immunotherapy in groups with high versus low IGFL1 expression, in addition to investigating correlations between IGFL1 expression and immune checkpoint markers. Genetic alterations of IGFL1 were analysed via cBioPortal, while the TIMER2.0 database was used to explore relationships between IGFL1 expression and key gene mutations in CRC. The CTRP and GDSC databases were employed to investigate associations between IGFL1 expression and sensitivity to conventional chemotherapy drugs. Finally, phenotypic validation and mechanistic studies were conducted using the CRC cell lines SW620 and HCT116.

**Results:**

Our study demonstrates that IGFL1 expression is significantly up-regulated in CRC and possesses considerable diagnostic value. Elevated IGFL1 levels were consistently observed in clinical specimens, where high expression correlated with adverse clinicopathological features, poorer prognosis, and mutations in key oncogenes. Within the tumour microenvironment, IGFL1 appears to play a critical role in modulating the infiltration of diverse immune cell populations. Furthermore, IGFL1 expression influences both immunotherapy responsiveness and chemotherapy sensitivity in CRC patients. Genetic knockdown of IGFL1 markedly attenuated the malignant phenotype of CRC cells. RNA-sequencing analysis revealed that IGFL1 is closely linked to cholesterol metabolism, autophagy pathways, and ATP hydrolysis activity. Functionally, inhibition of IGFL1 enhanced lipophagy in CRC cells. Collectively, these findings indicate that IGFL1 promotes CRC pathogenesis and progression through the suppression of lipophagy.

**Conclusions:**

IGFL1 exhibits oncogenic properties in colorectal cancer and may represent a potential therapeutic target.

## 1 Introduction

Colorectal cancer (CRC) persists as one of the most common malignancies worldwide (1, 2). Despite the implementation of multimodal treatment strategies—encompassing surgical resection, chemotherapy, radiotherapy, immunotherapy, and targeted therapies—CRC continues to represent the second leading cause of cancer-related mortality globally (3–5). Molecular biomarkers in oncology serve as quantifiable indicators that aid in accurate diagnosis, risk stratification, prediction of treatment response, and prognostic evaluation. Notably, many emerging targeted therapeutic agents are effective only in patients harbouring specific molecular alterations (6, 7). Consequently, the discovery of novel CRC tumour markers with high specificity and sensitivity remains essential to improving clinical management and patient outcomes.

The proteins encoded by IGFL-like (IGFL) genes are approximately 100 amino acids in length and are characterized by 11 cysteine residues occupying conserved positions, including two CC motifs (8). Initially, IGFL1 was identified as playing a specific role in certain dermatological disorders (9–11). More recent studies, however, increasingly associate IGFL1 overexpression with tumor-promoting activity across multiple cancer types, where it has been strongly linked to poor clinical outcomes and adverse biological behaviour (12, 13). For example, IGFL1 overexpression has been shown to promote tumourigenesis and progression in lung adenocarcinoma, establishing it as a predictor of unfavourable prognosis (14). Nevertheless, its expression profile in colorectal cancer (CRC) and the underlying mechanisms remain unclear.

This study systematically evaluates the multifaceted roles of IGFL1 in CRC using integrated bioinformatic analysis and *in vitro* experimentation, with particular focus on its influence on biological processes, tumour immunology, chemosensitivity profiles, and prognostic relevance. Furthermore, the investigation elucidates the specific mechanisms through which IGFL1 contributes to CRC progression and assesses its potential as a promising therapeutic target.

## 2 Materials and methods

### 2.1 Collection of data

This study utilized mRNA expression data alongside clinical data from colorectal cancer patients, all derived from the TCGA (The Cancer Genome Atlas) and GEO (Gene Expression Omnibus) databases. For normalization, standardization, and visualizations, the ‘limma’ and additional R (v3.6.3) packages were utilized (15).

### 2.2 Differential expression analysis

IGFL1 mRNA expression levels were compared between normal and tumour tissues. Following the removal of duplicate and incomplete RNA-seq entries from the GEO and TCGA databases, the data were normalised by log_2_ (TPM + 1) transformation. Differential expression of IGFL1 in colorectal cancer (CRC) was subsequently evaluated using the Wilcoxon rank-sum test. Prior to this analysis, normality of the IGFL1 expression data—in both paired and unpaired samples—was assessed via the Shapiro–Wilk test. Additionally, correlations between IGFL1 expression and clinical characteristics in the TCGA-COADREAD dataset were examined using the chi-square test. For all statistical tests, a p-value of < 0.05 was considered statistically significant.

### 2.3 Diagnostic receiver operating characteristic analysis

Receiver operating characteristic (ROC) analysis is a well-established statistical method used to assess the performance of diagnostic biomarkers. The ROC curve plots the true positive rate (sensitivity) against the false positive rate (1 – specificity) across a range of classification thresholds, with the relationship between these measures analysed quantitatively. A central summary measure derived from this analysis is the area under the curve (AUC), obtained by integrating sensitivity and specificity over all possible threshold values. In this study, ROC curves were generated using the ‘pROC’ package (version 3.50.0) in R, allowing precise evaluation of the discriminatory ability of candidate biomarkers and their potential clinical utility. The AUC value ranges from 0.5, representing classification no better than chance, to 1.0, indicating perfect discrimination; higher values denote superior diagnostic performance (16).

### 2.4 Survival analysis

Based on the median mRNA expression level of IGFL1, colorectal cancer patients were categorised into high- and low-expression groups. Subsequently, the association between IGFL1 expression status and key prognostic endpoints—namely Disease-Specific Survival (DSS) and Progression-Free Interval (PFI)—was evaluated.

### 2.5 Establishment and validation of the nomogram

To construct a prognostic model, multivariate Cox regression analysis was conducted, and the results were used to develop a nomogram that generated a continuous risk score. The prognostic accuracy of this score for predicting patient survival at 1, 3, and 5 years was evaluated by means of time-dependent ROC curves, while the score was retained in its continuous form without applying any cut-off threshold. Clinically relevant variables, including TNM stage, perineural invasion, lymphatic invasion, and IGFL1 expression, were obtained from TCGA database. The performance of the model was further validated using calibration plots and time-dependent ROC analysis.

### 2.6 Immune infiltration analysis

We utilised the TIMER2.0 database to obtain data on immune cell infiltration. Immune infiltration scores were calculated using CIBERSORT and ssGSEA methodologies, enabling subsequent correlation analysis with IGFL1 expression (17, 18). Finally, the ESTIMATE algorithm was applied to compare stromal scores, immune scores, and ESTIMATE scores between high and low IGFL1 expression groups (19).

### 2.7 Immune correlation analysis

RNA-seq raw read counts data and matched clinical information for colorectal cancer were obtained from TCGA database. Transcripts per million (TPM) values were then derived and normalised via log_2_(TPM + 1) transformation. Samples with both RNA-seq data and complete clinical annotations were included for subsequent analysis. The association between IGFL1 expression levels and immune cell infiltration was assessed using the immunedeconvR software package. Visualisation of results was carried out with the R package ggClusterNett (version 3.50.0) (20).

### 2.8 Single-cell sequencing analysis

Single-cell RNA sequencing data in. h5 format, along with associated annotation files, were obtained from the Tumor Immune Single-cell Hub (TISCH) database. Data processing and downstream analysis were conducted using the MAESTRO and Seurat packages within the R statistical environment. Subsequent cell clustering was performed by applying t-distributed stochastic neighbor embedding (t-SNE) for dimensionality reduction and visualisation (21).

### 2.9 Immune checkpoint

Immune checkpoints, which comprise cell-surface molecules expressed on immune cells, serve to modulate the degree of immune activation and prevent excessive responses. To examine potential differences in this regulatory pathway, we analysed and compared the expression profiles of several common immune checkpoint genes (ICGs) between the two study groups (22).

### 2.10 Genetic alteration analysis

Using the cBioPortal database, we assessed the mutation frequency and specific mutation sites of IGFL1 in CRC (23). The association between IGFL1 expression and mutations in key CRC−related genes was further evaluated using chi−square tests. The Genomic Data Commons (GDC) database provided all mutation data for TCGA samples (24).

### 2.11 Drug sensitivity analysis

The relationship between IGFL1 expression and sensitivity to conventional chemotherapeutic agents was investigated. RNA-Seq data along with corresponding clinopathological data for CRC were retrieved from TCGA database. The predicted chemotherapy response for each sample was then evaluated using drug sensitivity information from the Genomics of Drug Sensitivity in Cancer (GDSC) and the Cancer Therapeutics Response Portal (CTRP) databases (25, 26). Sample−specific drug sensitivity predictions, quantified as the half−maximal inhibitory concentration (IC50), were derived using the pRRophetic package in R. The analysis applied ridge regression to estimate IC50 values, with all computational parameters kept at their default configurations. Prior to analysis, gene expression data were preprocessed by correcting for batch effects using the ‘ComBat’ algorithm and adjusting for tissue type across all samples. Duplicate gene expression values were subsequently aggregated by taking the mean expression level.

### 2.12 Immunohistochemical

The human tissue samples utilised in this study were obtained from surgical resection specimens of patients diagnosed with CRC. The cohort consisted of 50 pairs of CRC tissues and their matched adjacent normal intestinal mucosal tissues. After collection, all tissues were fixed in 4% paraformaldehyde and subsequently embedded in paraffin. Sections from paraffin-embedded blocks were subjected to dewaxing, antigen retrieval, and blocking procedures. Thereafter, sections were incubated overnight at 4°C with a polyclonal antibody against IGFL1 (Proteintech, China; Cat# 20545-1-AP; diluted 1:200). Following primary antibody incubation, sections were treated with a biotin-conjugated secondary antibody. Staining was visualised using 3,3’-diaminobenzidine (DAB) as the chromogen, followed by haematoxylin counterstaining. Images were acquired using an optical microscope. Importantly, all immunohistochemical experiments were independently repeated at least three times.

Immunohistochemical staining was evaluated independently by three pathologists, each of whom separately scored staining intensity (I) and the proportion of positively stained cells (S). The percentage score (S) was recorded on a scale of 0–100%, while staining intensity (I) was graded on a scale of 0–3, representing absent, weak, moderate, and strong staining, respectively. All assessments were performed through systematic microscopic examination of each field of view. An immunostaining score (IS) was then derived for each case by multiplying the percentage score (S) by the intensity grade (I), resulting in a possible score range of 0–3.

Prior to the commencement of the study, ethical approval was granted by the Institutional Review Board (IRB) of the Affiliated Hospital of Panzhihua University. All study procedures were conducted in strict accordance with the most recent version of the Declaration of Helsinki. In addition, written informed consent was obtained from each participating patient prior to enrolment.

### 2.13 Cell culture

Both cell lines were obtained from the Cell Resource Centre of the Chinese Academy of Medical Sciences. Cells were cultured in Dulbecco’s Modified Eagle Medium (DMEM; Gibco, USA) supplemented with 4.5 g/L glucose, 10% fetal bovine serum (FBS), and 1% penicillin/streptomycin (Gibco, USA).

### 2.14 Lentivirus infection and cell transfection

To generate stable IGFL1 knockdown in CRC cell lines (SW620 and HCT116), lentiviral-mediated shRNA delivery was performed. Lentiviral vectors encoding short hairpin RNAs (shRNAs) targeting IGFL1 were transduced into the cells. Following infection with the packaged lentiviral particles, stable polyclonal cell populations were selected using puromycin to ensure consistent IGFL1 knockdown.

### 2.15 Quantitative reverse transcription PCR

Total RNA was extracted from cultured cells and reverse-transcribed into complementary DNA (cDNA). Target sequences were subsequently amplified by quantitative real-time polymerase chain reaction (qRT−PCR). Gene expression levels were quantified using the comparative cycle threshold (Ct) method and calculated via the 2^−ΔΔCt formula.

### 2.16 Western blot analysis

Protein samples (40 µg per lane) were extracted from cells and separated by electrophoresis on 10% SDS-polyacrylamide gels. Following separation, proteins were electroblotted onto a PVDF membrane. The membrane was blocked with 5% non-fat milk to minimise non-specific binding and subsequently incubated overnight at 4°C with the appropriate primary antibody. After three washes with PBST, the membrane was incubated for two hours at room temperature with an HRP-conjugated secondary antibody. A final series of three PBST washes was performed to remove any unbound antibody. Protein bands were quantified by densitometry using ImageJ software and normalised to the loading control, GAPDH.

The following primary antibodies were used: CD44 polyclonal antibody (Proteintech, China; Cat# 15675-1-AP; dilution 1:6000), SOX2 polyclonal antibody (Proteintech, China; Cat# 11064-1-AP; dilution 1:600), OCT4/POU5F1 polyclonal antibody (Proteintech, China; Cat# 11263-1-AP; dilution 1:2500), P62/SQSTM1 polyclonal antibody (Proteintech, China; Cat# 18420-1-AP; dilution 1:10000), LC3 polyclonal antibody (Proteintech, China; Cat# 14600-1-AP; dilution 1:6000), and GAPDH polyclonal antibody (Proteintech, China; Cat# 10494-1-AP; dilution 1:20000).

### 2.17 Cell viability assay

Cells were seeded in 96-well plates at a density of 2,000 cells per well and cultured for 48 hours. Following this incubation, 10 μL of CCK-8 reagent was added to each well and the plates were further incubated at 37 °C for 2 hours. Absorbance at 450 nm was then measured using a Thermo Fisher Scientific Multiskan Spectrum microplate reader. Cell viability was calculated according to the following formula: [(OD of test group − blank)/(OD of control group − blank)] × 100%.

### 2.18 Colony formation test

Two cell lines were uniformly plated in six-well plates at a density of 200 cells per well. After visible colony formation, the cells were fixed with methanol and stained with 10% Giemsa solution. Colonies were then imaged and quantified.

### 2.19 Invasion test

A cell suspension containing 5 × 10^4^ cells in 100 µL of serum-free DMEM medium was seeded into the upper chamber of a Transwell insert. The lower chamber was filled with 600 µL of complete medium supplemented with 10% FBS. After assembly, the Transwell plate was incubated at 37 °C for 48 h. Following incubation, invasive cells on the lower surface of the membrane were fixed and stained with 10% Giemsa solution. Images were captured using a light microscope for subsequent analysis.

### 2.20 Wound-healing assay

Cells were seeded into six-well plates at a density of 5×10^5^ cells per well. A straight scratch was then introduced in each well using a 200 µL pipette tip by gentle scraping. Following scratching, the wells were washed with phosphate-buffered saline (PBS) to remove detached or non-adherent cells. Images were captured at a single time point (24 h post-scratch) using a digital camera attached to a Motic light microscope.

### 2.21 Spheroid formation assay

Two hundred microlitres of serum-free DMEM medium was dispensed into each well of a 96-well plate at a seeding density of 200 cells per well, with six replicate wells per experimental group. Cells were maintained under standard culture conditions (37 °C, 5% CO_2_) for a period of two weeks. By day 14, well-defined cellular spheroids had formed. The culture medium was replenished every four days with freshly prepared complete stem cell medium; medium changes were performed once the spheroids had developed into floating spherical colonies. Spheroid formation was quantified by imaging five randomly selected microscopic fields per group.

### 2.22 Analysis of apoptosis

Cells were fixed in 4% paraformaldehyde for 30–60 minutes. Subsequently, 100 μl of TUNEL assay reagent was added to each sample, followed by incubation at 37 °C for 60 minutes in the dark. After incubation, samples were washed three times with PBS. Finally, samples were mounted using an antifade mounting medium and observed under a fluorescence microscope.

### 2.23 mRNA sequencing

Total RNA was extracted from cultured cells using the RNeasy Mini Kit (Qiagen, Manchester, UK) in accordance with the manufacturer’s instructions. Genomic DNA was removed by on-column DNase I digestion. RNA integrity was assessed using the Agilent 4200 TapeStation System (Agilent Technologies, Cheadle, UK); all samples exhibited an RNA Integrity Number (RIN) above 8.0. RNA concentration and purity were determined with a NanoDrop™ One spectrophotometer (Thermo Fisher Scientific, Loughborough, UK), confirming that the A260/280 and A260/230 ratios fell within acceptable limits.

Sequencing libraries were prepared from 1 µg of high-quality total RNA per sample using the NEBNext^®^ Ultra™ II Directional RNA Library Prep Kit for Illumina^®^ (New England Biolabs, Hitchin, UK). The protocol included poly-A-based mRNA enrichment, RNA fragmentation, first- and second-strand cDNA synthesis, adapter ligation, and PCR amplification. Final library quality and size distribution were verified on the Agilent 4200 TapeStation System (Agilent Technologies, Cheadle, UK), and quantification was performed by qPCR using the Kapa Library Quantification Kit (Roche, Burgess Hill, UK). Barcoded libraries were pooled in equimolar amounts and sequenced on an Illumina NovaSeq 6000 platform (Illumina, Cambridge, UK) to generate 150 bp paired-end reads, with a target depth of 40 million reads per sample.

Initial raw sequencing data in FASTQ format were subjected to quality control using FastQC (v0.11.9). Adapter sequences and low-quality bases were trimmed with Trim Galore (v0.6.7), which employs Cutadapt (v3.4) to remove bases with a Phred score < 20. High-quality reads were then aligned to the GRCh38 human reference genome using the splice-aware aligner STAR (v2.7.10a). Gene-level read counts were derived from uniquely mapped reads overlapping exons as defined in the GENCODE v35 annotation file, using featureCounts (v2.0.3) from the Subread package. Differential gene expression analysis was conducted with the DESeq2 package (v1.30.1) in R. Genes were considered significantly differentially expressed if they exhibited an adjusted p-value (Benjamini–Hochberg) below 0.05 and an absolute log2 fold change greater than 1.

### 2.24 Lipid droplet staining using Nile red

The cultured cells were washed twice with PBS and fixed with 4% (w/v) paraformaldehyde in PBS for 15 min at room temperature. After fixation, the cells were rinsed thoroughly with PBS. A working solution of Nile red (9-diethylamino-5H-benzo[α]phenoxazine-5-one; Sigma-Aldrich) was prepared by diluting the 1 mg/ml stock solution in acetone to a final concentration of 1 µg/ml in PBS. The fixed cells were then incubated with the Nile red working solution for 10 min in the dark at room temperature. Unbound dye was removed by washing the cells three times with PBS. The stained samples were examined and imaged using a confocal laser scanning microscope.

### 2.25 Lipid droplet staining using oil red O

Cells fixed in 4% paraformaldehyde were stained with a filtered Oil Red O working solution (0.3% in 60% isopropanol) for 30 minutes at room temperature. After incubation, the staining solution was aspirated, and the cells were washed extensively with distilled water to remove any non-specific staining. The stained samples were covered with an aqueous mounting medium and imaged immediately using a bright-field light microscope.

### 2.26 Transmission electron microscopy

Cell pellets were first fixed in 2.5% glutaraldehyde and then post-fixed in 1% osmium tetroxide. Following dehydration through a graded ethanol series, the samples were embedded in epoxy resin and prepared for ultrathin sectioning. The resulting sections were stained with uranyl acetate and lead citrate, and examined using a transmission electron microscope operating at 120 kV.

### 2.27 Immunofluorescence

Following a brief PBS wash, cells grown on glass coverslips were fixed with 4% (w/v) paraformaldehyde for 15 min at room temperature. Subsequently, cells were permeabilised and blocked by incubation in PBS containing 0.1% Triton X-100 and 5% normal serum for 60 min at room temperature to minimise non-specific antibody binding. Primary antibodies, diluted in an antibody dilution buffer, were applied to the samples and incubated overnight at 4 °C. The primary antibodies and dilutions used were: anti-LC3 monoclonal antibody (1:500; Proteintech, #66139-3-Ig) and BODIPY 493/503 (1:2500; Proteintech, #CM02294). After incubation, unbound primary antibodies were removed by washing the coverslips three times for 5 min each in PBS under gentle agitation. The samples were then incubated for 1 h at room temperature in the dark with fluorophore-conjugated secondary antibodies (Alexa Fluor 488, 594; Invitrogen) diluted in antibody dilution buffer. Following another three 5-minute washes with PBS, the coverslips were rinsed thoroughly in PBS and distilled water, and mounted onto glass microscope slides using a polyvinyl alcohol-based antifade mounting medium (Vectashield). Images were acquired using a Leica SP8 confocal laser scanning microscope system equipped with appropriate lasers and optical filters.

### 2.28 Statistical analysis

All statistical analyses were performed using GraphPad Prism (version 10.1.2) and R software. Between-group comparisons were carried out using two-tailed unpaired Student’s t-tests, Wilcoxon tests, or two-way ANOVA, as appropriate. Survival outcomes were assessed with the log-rank test. A p-value < 0.05 was considered statistically significant, with significance levels denoted as follows: *p < 0.05, **p < 0.01, ***p < 0.001, and ****p < 0.0001. All experiments were independently repeated at least three times.

## 3 Results

### 3.1 High expression of IGFL1 in CRC

**Figure 1:**
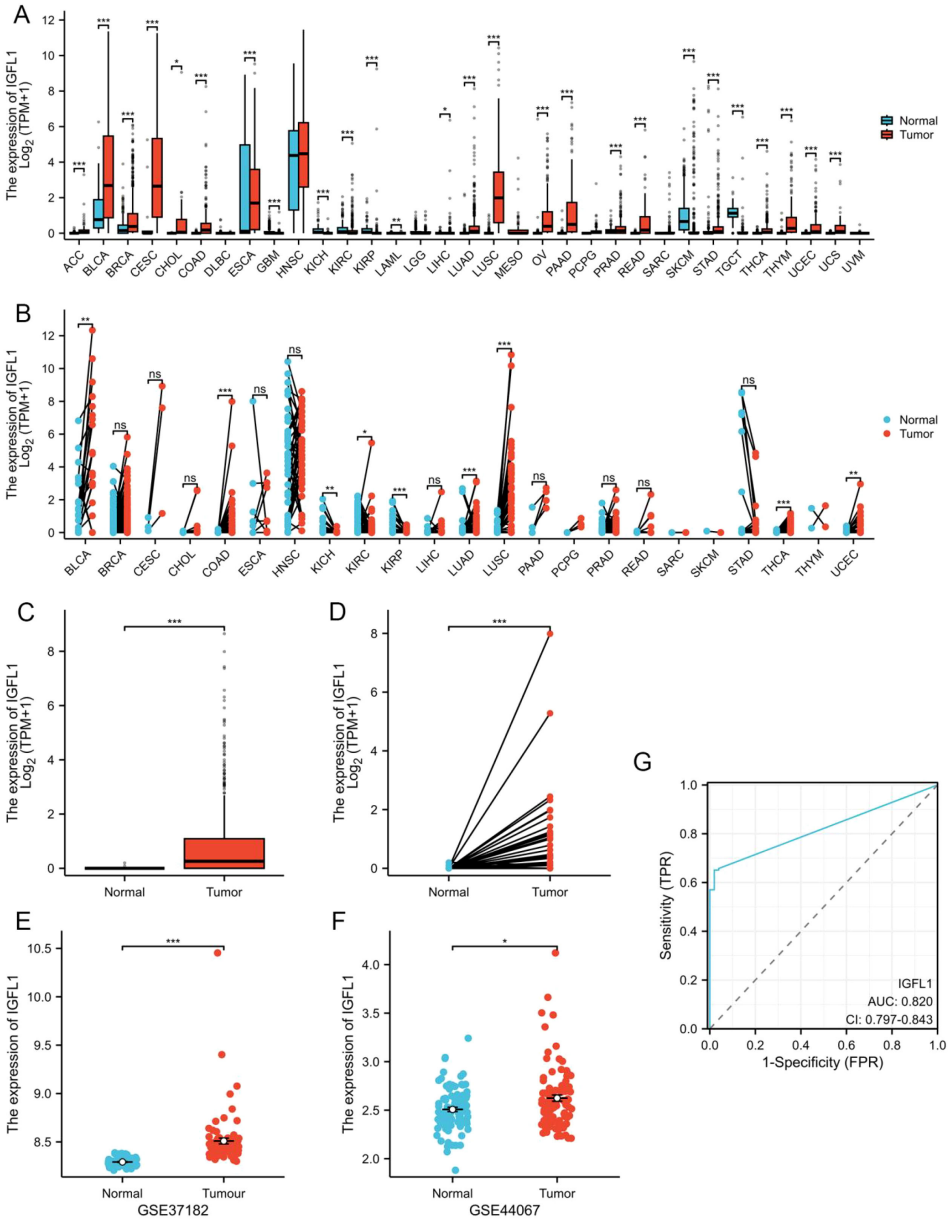
IGFL1 expression levels in tumor tissues. Analysis of IGFL1 transcript levels was conducted using data from **(A)** unpaired and **(B)** paired samples within the TCGA cohort. Similarly, expression profiles in **(C)** unpaired and **(D)** paired samples were derived from the TCGA-COADREAD dataset. Expression of IGFL1 in the **(E)** GSE37182 and **(F)** GSE44067 databases. Assessment of diagnostic potential revealed an area under the ROC curve (AUC) of 0.82 **(G)**, demonstrating that IGFL1 possesses significant value as a diagnostic biomarker for CRC. Statistical significance was determined using two-tailed unpaired Student’s t-tests (*p < 0.05, **p < 0.01, ***p < 0.001).

An initial comparative assessment of IGFL1 transcript levels across pan-cancer datasets revealed significant overexpression in 20 malignancies. As shown in **Figure 1A**, notably elevated expression was observed in colon adenocarcinoma (COAD; p < 0.001) and rectum adenocarcinoma (READ; p < 0.001). This upregulation pattern was further confirmed in paired tumour specimens, where IGFL1 expression was consistently increased across multiple cancer types including COAD (**Figure 1B**).

Furthermore, analysis of the TCGA-COADREAD cohort demonstrated significantly higher IGFL1 levels in tumour tissues compared with normal controls (p < 0.001), with this differential expression maintained in both paired (**Figure 1C**) and unpaired (**Figure 1D**) sample analyses. To validate the findings from the TCGA database, we utilised an independent external cohort obtained from GEO datasets. Following comprehensive evaluation, the GSE37182 and GSE44067 datasets were selected for verification. Consistent with the TCGA results, both datasets confirmed a significant upregulation of IGFL1 expression in colorectal cancer relative to adjacent normal tissues (**Figures 1E, F**).

In addition, the diagnostic accuracy of IGFL1 gene expression for CRC detection was assessed using ROC curve analysis. The resulting AUC value of 0.82 (**Figure 1G**) reflects a strong discriminatory capacity of IGFL1 in distinguishing CRC patients from healthy controls. These findings suggest the potential utility of IGFL1 as a diagnostic biomarker for CRC, which could facilitate earlier disease detection and improved clinical management.

**Figure 2:**
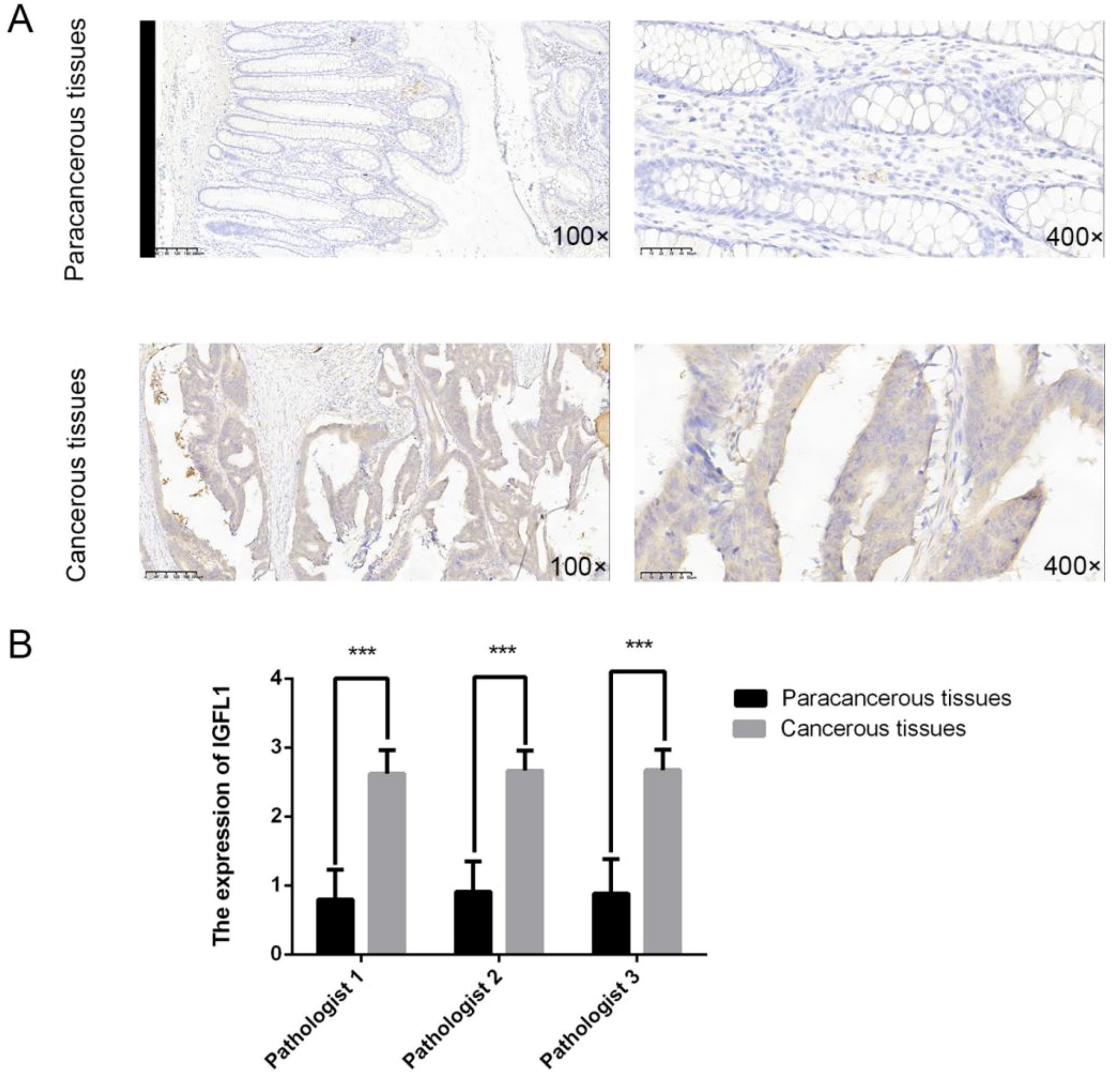
Confirmation of IGFL1 protein levels in clinical specimens via immunohistochemistry. **(A)** Representative photomicrographs depicting IGFL1 immunohistochemical staining in paired adjacent non-tumor and tumor tissues from CRC patients. **(B)** Comparative analysis of IHC staining intensity scores for IGFL1 in matched paracancerous and cancerous tissues derived from 50 CRC cases (Wilcoxon signed rank test, p < 0.001). *** means p < 0.001.

To further corroborate these observations, we performed immunohistochemical analysis on randomly selected tumour tissues and matched adjacent normal tissues from 50 colorectal carcinoma cases. Immunohistochemical staining indicated that IGFL1 was predominantly localised in the cytoplasm. Compared with adjacent normal tissues, IGFL1 expression was significantly upregulated in colorectal cancer tissues (**Figures 2A, B**).

### 3.2 Elevated IGFL1 expression is associated with adverse clinicopathological progression

**Figure 3:**
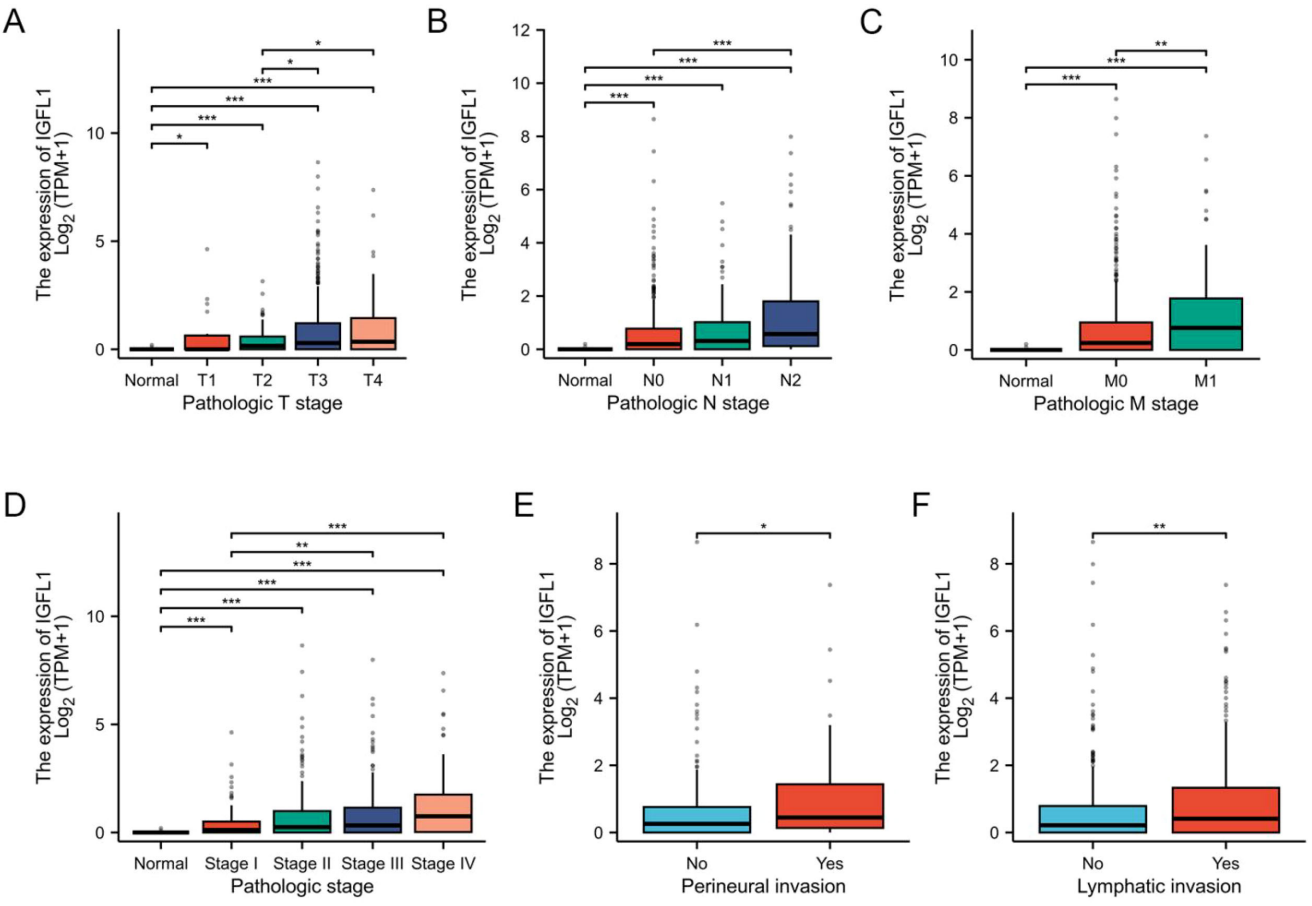
Correlation of IGFL1 expression levels with clinicopathological features from TCGA-COADREAD dataset. The figure presents data for the following parameters: caption is as follows: **(A)** pathological T stage, **(B)** pathological N stage, **(C)** pathological M stage, **(D)** pathological stage, **(E)** perineural invasion status, and **(F)** lymphatic invasion status. Statistical significance was determined using two-tailed unpaired Student’s t-tests (*p < 0.05, **p < 0.01, ***p < 0.001).

Analysis of the TCGA-COADREAD (colon and rectal cancer) cohort revealed that elevated IGFL1 expression was significantly associated with more advanced tumour stages. Specifically, higher expression was observed in T4 versus T2, N2 versus N0, and M1 versus M0 categories, as well as in Pathologic stage IV compared to Pathologic stage I (**Figures 3A–D**). Moreover, increased IGFL1 expression correlated with a higher incidence of perineural invasion and lymphatic metastasis (**Figures 3E, F**). These findings suggest that evaluating IGFL1 expression could be integrated into clinicopathological diagnostics, potentially enabling more accurate pathological staging.

### 3.3 High expression of IGFL1 is associated with poor prognosis

**Figure 4:**
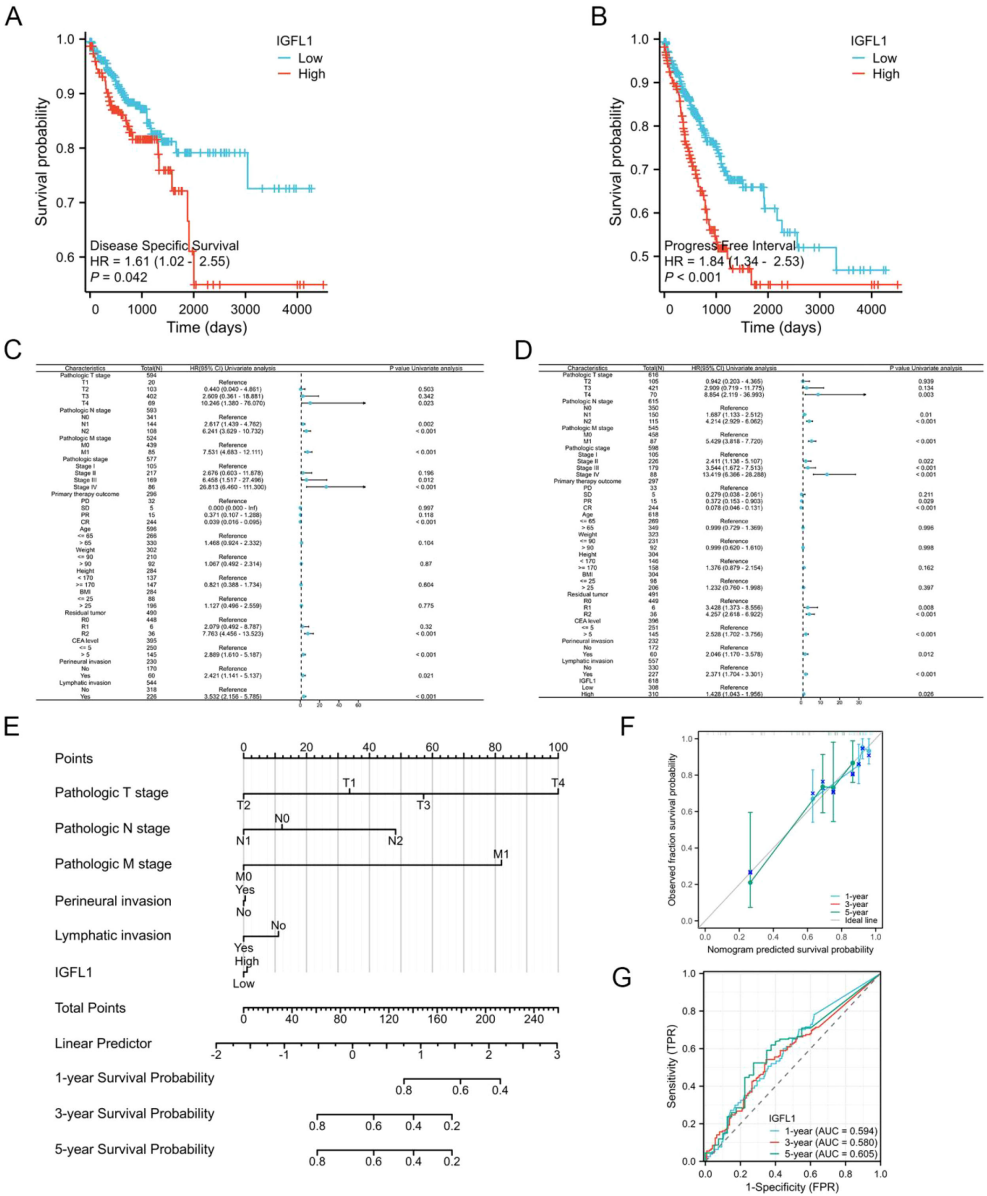
In TCGA-COADREAD, Cox regression analysis and Kaplan-Meier analysis were employed to evaluate the prognostic value of IGFL1 expression levels and to construct a prognostic model. Panels **(A, B)** illustrate DSS and PFI curves, respectively, comparing cohorts stratified by high versus low IGFL1 expression. Univariate Cox proportional hazards regression analysis yielded forest plots depicting hazard ratios for DSS **(C)** and PFI **(D)** in the CRC cohort. **(E)** A nomogram for predicting 1-, 3- and 5-year PFI survival of CRC. **(F)** The calibration curve for the 1-, 3- and 5-year PFI survival nomogram. **(G)** ROC curves for the nomogram model at 1-, 3- and 5-year.

Compared with patients exhibiting low IGFL1 expression, those with high IGFL1 expression showed significantly poorer disease-specific survival (DSS; hazard ratio [HR] = 1.61, p = 0.042) and progression-free interval (PFI; HR = 1.84, p < 0.001) (**Figures 4A, B**).

To comprehensively assess prognostic factors related to IGFL1 expression, we performed univariate Cox regression analyses. Several clinical characteristics emerged as independent determinants influencing either DSS or PFI. In univariate analysis of CRC patients, independent risk factors for DSS included T4 stage, nodal involvement (N1 or N2), distant metastasis (M1 stage), advanced pathologic stage (III or IV), achievement of complete response (CR) to primary therapy, presence of macroscopic residual tumour (R2), serum CEA level > 5 ng/mL, and the presence of perineural or lymphatic invasion (**Figure 4C**).

For PFI, independent predictors included elevated IGFL1 expression, advanced T stage (T4), nodal involvement (N1, N2), distant metastasis (M1), higher pathologic stages (II, III, IV), favourable primary therapy outcomes (partial response [PR] or complete response [CR]), residual tumour (R1, R2), elevated CEA level (> 5 ng/mL), and the presence of perineural or lymphatic invasion (**Figure 4D**).

Subsequent multivariate Cox regression analysis of these significant variables identified T3 and T4 stages, N1 stage, M1 stage, perineural invasion, and lymphatic invasion as independent prognostic factors for DSS (**Table 1**). For PFI, N1 stage, pathologic stage II, male sex, and height ≥ 170 cm were independently associated with outcome (**Table 2**).

**Table 1:** Multivariate Cox regression analyses of clinical characteristics associated with DSS of CRC.

Characteristics	Total(N)	HR(95% CI) Multivariate analysis	P value Multivariate analysis
Pathologic T stage	618		
T1	20	Reference	
T2	109	0.000 (0.000 - Inf)	0.999
T3	416	0.000 (0.000 - 0.000)	< 0.001
T4	73	0.000 (0.000 - 0.000)	< 0.001
Pathologic N stage	617		
N0	358	Reference	
N1	147	109546293545.5090 (8341263103.2223 - 1438677845436.0100)	< 0.001
N2	112	3705290819293339984045522550784.0000 (0.000 - Inf)	0.998
Pathologic M stage	542		
M0	455	Reference	
M1	87	0.000 (0.000 - 0.000)	< 0.001
Perineural invasion	233		
No	173		
Yes	60		< 0.001
Lymphatic invasion	568		
No	337		
Yes	231		< 0.001

**Table 2:** Multivariate Cox regression analyses of clinical characteristics associated with PFI of CRC.

Characteristics	Total(N)	HR(95% CI) Multivariate analysis	P value Multivariate analysis
Pathologic N stage	639		
N0	367	Reference	
N1	153	9542128383727.8008 (572460461427.5580 - 159054153477228.0000)	< 0.001
N2	119	572496902.3052 (0.000 - Inf)	0.997
Pathologic stage	622		
Stage I	111	Reference	
Stage II	237	9433776533731026.0000 (565960186562286.0000 - 157248057021374656.0000)	< 0.001
Stage III	184	1.000 (0.060 - 16.669)	1
Stage IV	90	1.000 (0.000 - Inf)	1
Gender	643		
Female	301	Reference	
Male	342	0.000 (0.000 - 0.000)	< 0.001
Height	329		
< 170	159	Reference	
>= 170	170	4872249013645.7402 (292300675473.8670 - 81213669494561.7969)	< 0.001

Based on these results, we developed a prognostic nomogram incorporating TNM stage, perineural invasion, lymphatic invasion, and IGFL1 expression, which demonstrated significant predictive ability for clinical outcomes (**Figure 4E**). A calibration plot revealed good agreement between predicted and observed values, with the bias-corrected line closely approximating the ideal 45° reference line (**Figure 4F**). Moreover, time-dependent receiver operating characteristic (ROC) analysis yielded AUC values of 0.594, 0.580, and 0.605 for 1-, 3-, and 5-year survival, respectively (**Figure 4G**).

### 3.4 High expression of IGFL1 influences the immune microenvironment and immune score

**Figure 5:**
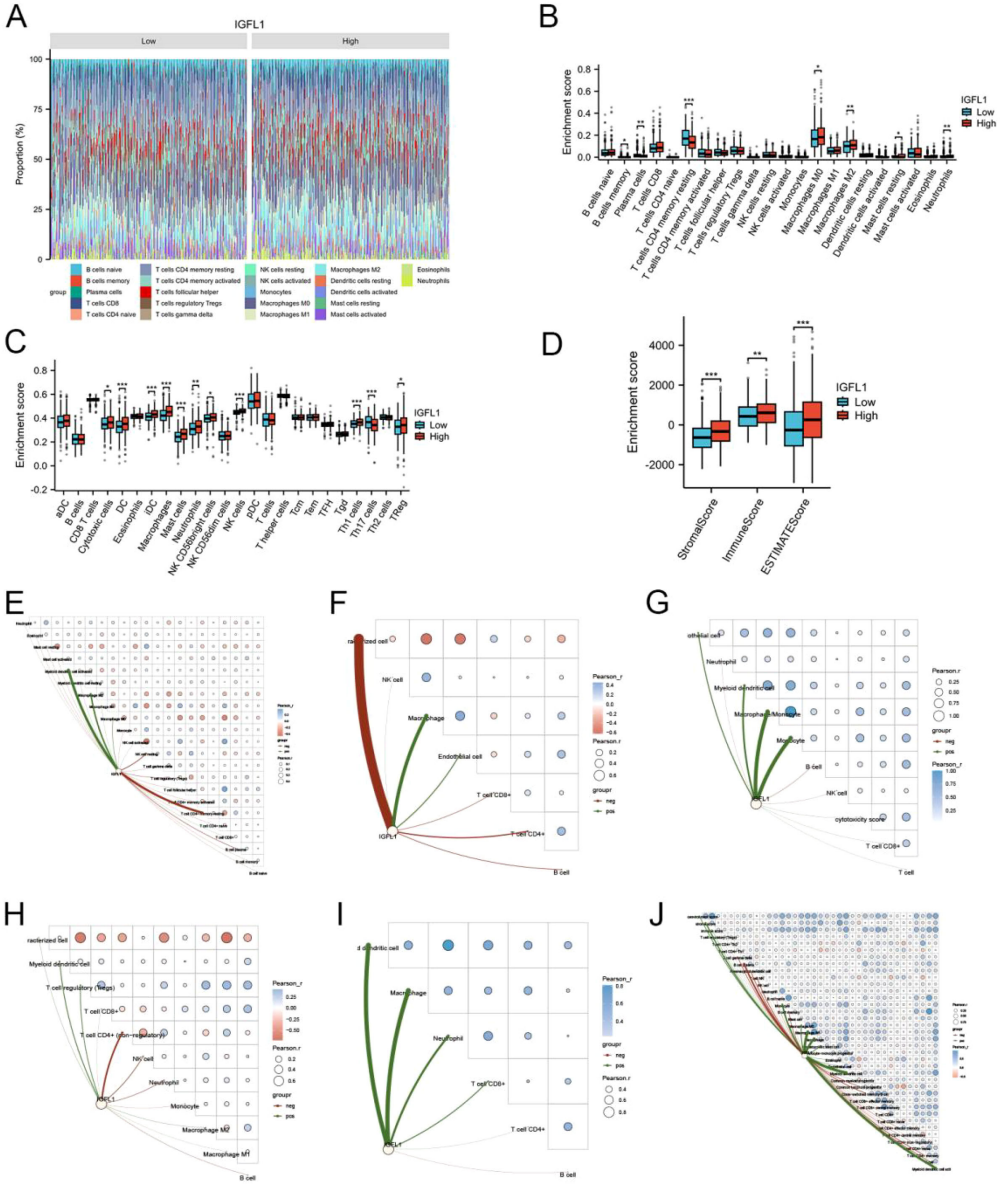
Assessment of immune infiltration between IGFL1 high- and low-expression groups. Differences in the proportions of various immune cell types infiltrating the two groups were assessed using the Cibersort **(A, B)** and ssGSEA **(C)** algorithms. Differences in immune scores between the two groups were assessed using the estimate algorithm **(D)**. In TCGA-COADREAD, the correlation between IGFL1 expression and immune scores was evaluated using six state-of-the-art algorithms: CIBERSOR **(E)**, EPIC **(F)**, MCP-counter **(G)**, quanTIseq **(H)**, TTIMER **(I)**, and xCell **(J)**. * means p < 0.05**, means p < 0.01***, means p < 0.001.

Subsequently, we applied the CIBERSORT, ssGSEA, and ESTIMATE algorithms to further examine the infiltration levels of distinct immune cell types and immune scores, stratified by IGFL1 expression. First, CIBERSORT analysis revealed statistically significant differences (P < 0.05; **Figures 5A, B**) between the high- and low-IGFL1 expression groups in the infiltration levels of plasma cells, resting memory CD4^+^ T cells, M0 macrophages, M2 macrophages, resting mast cells, and neutrophils. Second, ssGSEA results indicated significant differences (P < 0.05; **Figure 5C**) between the groups for the following immune cell subsets/signatures: cytotoxic cells, dendritic cells (DCs), immature dendritic cells (iDCs), macrophages, mast cells, neutrophils, CD56^bright^ natural killer cells (NK CD56^bright^ cells), natural killer cells (NK cells), T helper 1 cells (Th1 cells), T helper 17 cells (Th17 cells), and regulatory T cells (Tregs). Finally, ESTIMATE algorithm analysis showed that the high IGFL1 expression group exhibited significantly higher stromal scores, immune scores, and ESTIMATE scores compared to the low expression group (P < 0.05; **Figure 5D**).

To further investigate the influence of IGFL1 expression on the colorectal cancer (CRC) immune microenvironment, we employed six established algorithms to evaluate the correlation between IGFL1 expression and immune cell scores. The results demonstrated that, under the CIBERSORT algorithm, IGFL1 expression was significantly negatively correlated with resting CD4^+^ memory T cells and resting NK cells, while showing a significant positive correlation with M2 macrophages and activated myeloid dendritic cells (**Figure 5E**). Using the EPIC algorithm, IGFL1 expression correlated negatively with uncharacterised cells and CD4^+^ T cells, and positively with macrophages and endothelial cells (**Figure 5F**). With the MCPCOUNTER algorithm, IGFL1 expression was significantly negatively correlated with B cells and NK cells, but positively correlated with macrophage/monocyte and monocyte populations (**Figure 5G**). Under the QUANTISEQ algorithm, a significant negative correlation was observed with CD4^+^ T cells and NK cells, whereas a positive correlation was found with myeloid dendritic cells and regulatory T cells (Tregs) (**Figure 5H**). The TIMER algorithm indicated a significant negative correlation with B cells and a positive correlation with myeloid dendritic cells and macrophages (**Figure 5I**). Finally, using the XCELL algorithm, IGFL1 expression correlated negatively with plasma B cells and NK T cells, but positively with myeloid dendritic cells and activated myeloid dendritic cells (**Figure 5J**).

**Figure 6:**
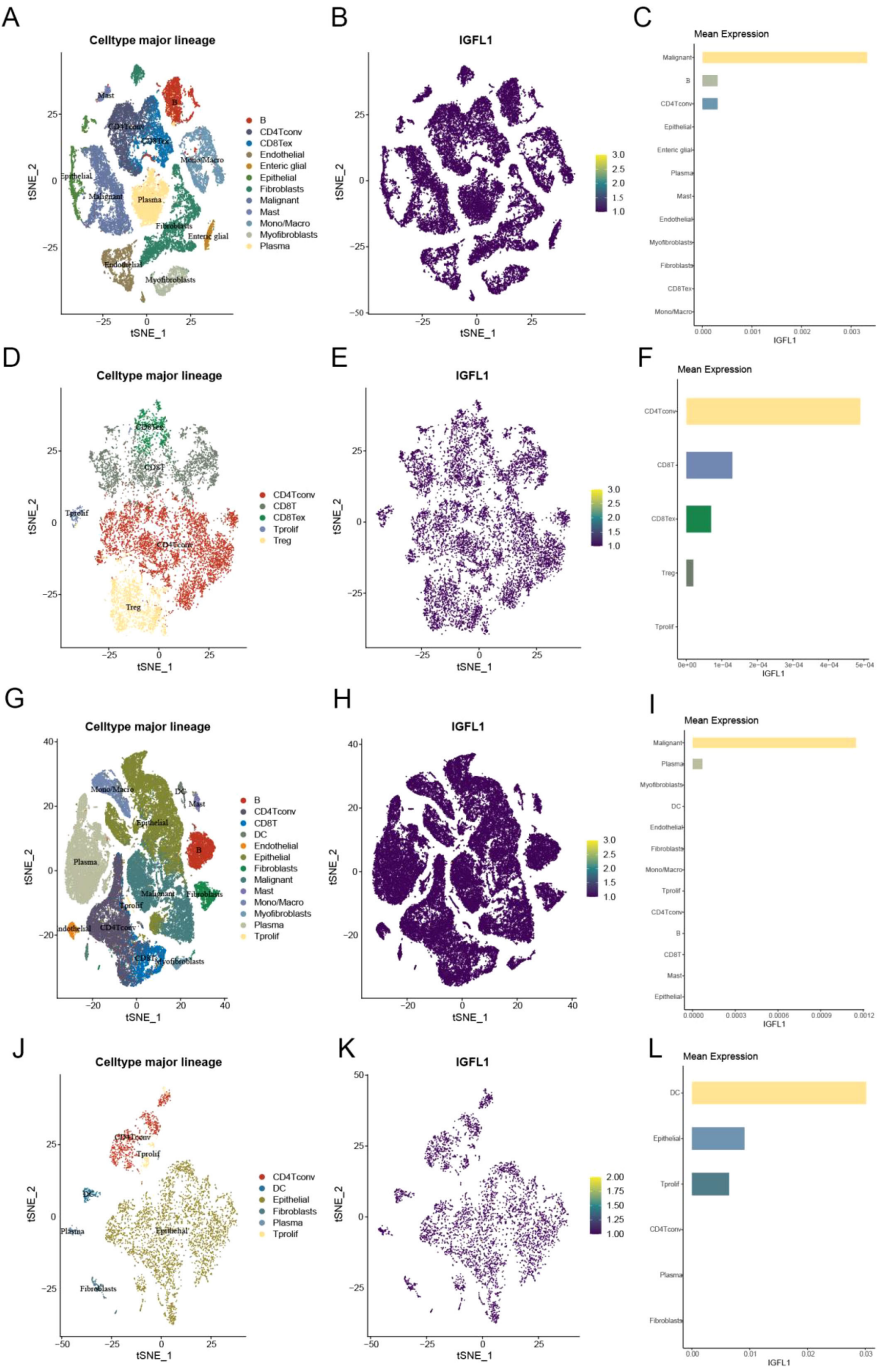
Expression levels of IGFL1 across cellular subpopulations were analyzed using four single-cell datasets from CRC. **(A–C)** EMTAB-8107 dataset; **(D–F)** GSE108989 dataset; **(G–I)** GSE166555 dataset; **(J–L)** GSE179784 dataset.

To validate these immune infiltration findings from the TCGA database, we analysed four single-cell RNA sequencing datasets from TISCH. In dataset EMTAB-8107, IGFL1 was co-expressed with malignant cells, B cells, and conventional CD4^+^ T cells (CD4Tconv) (**Figures 6A–C**). In GSE108989, IGFL1 co-expression was observed with CD4Tconv, CD8^+^ T cells, and exhausted CD8^+^ T cells (CD8Tex) (**Figures 6D–F**). In GSE166555, IGFL1 was co-expressed with malignant cells and plasma cells (**Figures 6G–I**). In GSE179784, co-expression was detected with dendritic cells (DCs), epithelial cells, and proliferating T cells (Tprolif) (**Figures 6J–L**). Collectively, these results suggest a strong association between an activated immune state within the tumour and high IGFL1 expression.

### 3.5 IGFL1 overexpression reduces immunotherapy efficacy

**Figure 7:**
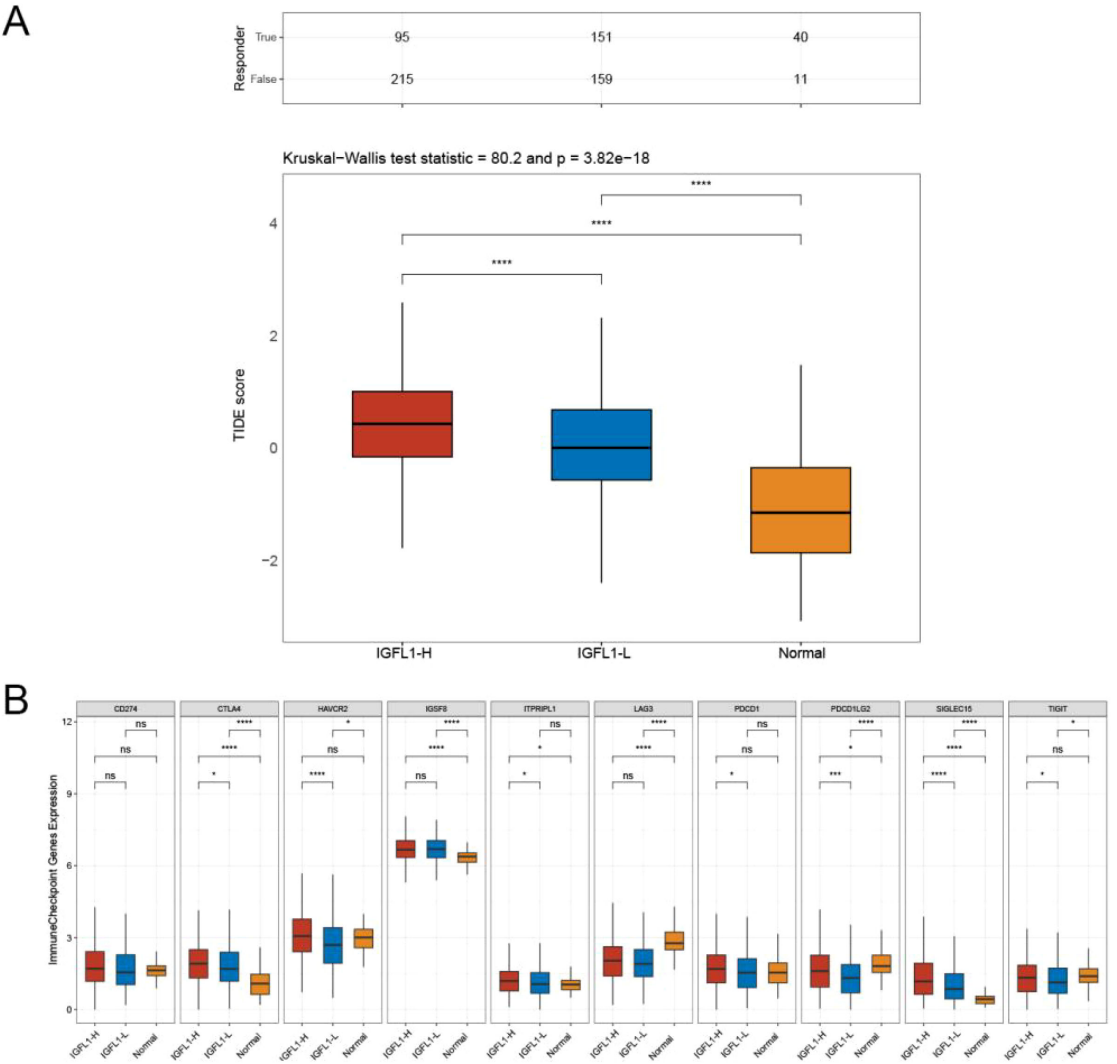
Relation between IGFL1 and TIDE and immune checkpoint expression. **(A)** The TIDE prediction algorithm assessed the differences in response to immunotherapy between the high and low IGFL1 expression groups. **(B)** Expression of different immune checkpoints in the high and low IGFL1 expression groups. * means p < 0.05,*** means p < 0.001****, means p < 0.0001.

Using the TIDE algorithm, we evaluated the potential clinical implications of immunotherapy in groups stratified by IGFL1 expression levels. Higher TIDE prediction scores are associated with a lower likelihood of clinical benefit from immune checkpoint inhibitor (ICI) therapy, reflecting an increased probability of tumour immune evasion. Our analysis revealed that the high IGFL1 expression cohort exhibited significantly elevated TIDE scores compared to the low-expression cohort, whereas the low IGFL1 expression group showed a greater frequency of positive responses to immunotherapy (**Figure 7A**). Additionally, we compared the expression profiles of immune checkpoint molecules between IGFL1 high- and low-expression groups. Significant differences were observed in the expression levels of CTLA4, HAVCR2, ITPRIPL1, PDCD1, PDCD1LG2, SIGLEC15, and TIGIT between the two cohorts (**Figure 7B**).

### 3.6 Genetic alteration of IGFL1 in CRC

**Figure 8:**
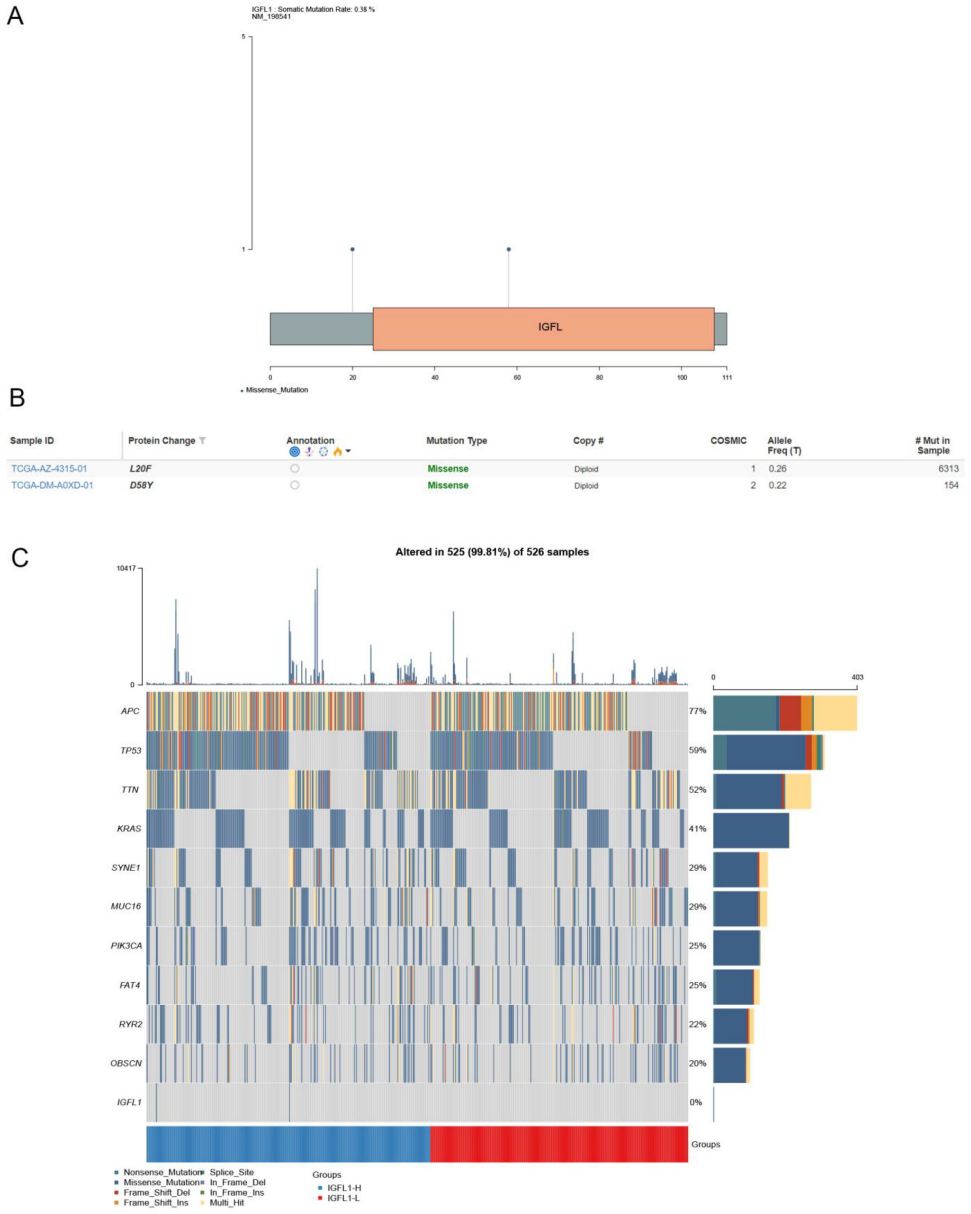
Genetic alterations of IGFL1 in CRC and their relationship to mutations in other key genes. **(A)** Diagram showing IGFL1 mutations across protein domains in CRC, derived from cBioPortal data. **(B)** IGFL1 genetic alterations in CRC, based on data from the cBioPortal database. **(C)** Mutation landscape of key genes stratified by high and low IGFL1 expression in CRC.

Drawing upon a dataset of 526 colorectal cancer samples with comprehensive genomic profiles obtained from the cBioPortal database, we evaluated the prevalence and spectrum of IGFL1 mutations. Analysis revealed two missense mutation sites within the amino acid region spanning positions 0 to 111, specifically L20F and D58Y. The overall mutation frequency was determined to be 0.38% (**Figures 8A, B**).

### 3.7 Elevated expression of IGFL1 is associated with a higher frequency of genetic mutations

Comparative analysis of genomic mutation profiles in colorectal cancer (CRC) patients with high versus low IGFL1 expression revealed substantial differences (**Figure 8C**). In particular, mutation frequencies of key genes—including APC, TP53, TTN, KRAS, and SYNE1—differed markedly between the two groups. Notably, APC and TP53, both established drivers of poor prognosis in CRC, showed significantly higher mutation rates in patients with elevated IGFL1 expression. These observations suggest a potential association between increased IGFL1 expression and colorectal cancer progression.

### 3.8 Overexpression of IGFL1 confers resistance to the majority of conventional chemotherapeutic agents

**Figure 9:**
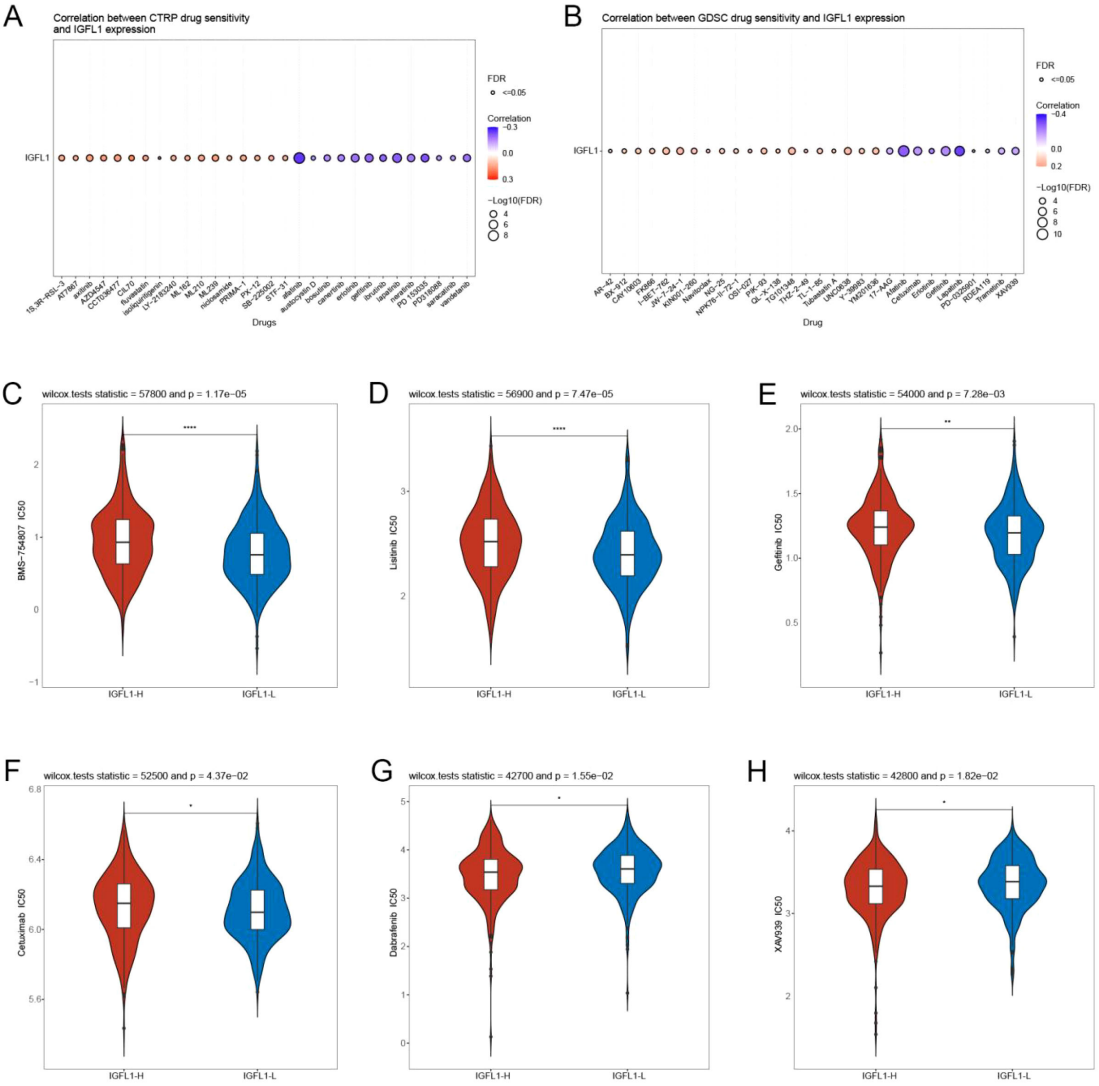
Drug sensitivity analysis of IGFL1in CRC. Relationship between CTRP **(A)** and GDSC **(B)** drug sensitivity and G6PD expression in CRC. Comparison of IC50 values for common chemotherapeutic drugs between high and low IGFL1 expression groups in CRC: **(C)** BMS-754807, **(D)** Linsitinib, **(E)** Gefitinib, **(F)** Cetuximab, **(G)** Dabrafenib, and **(H)** XAV939. (*P < 0.05; **P < 0.01; ****P < 0.0001).

Utilising the CTRP and GDSC databases, we evaluated the association between IGFL1 expression levels and sensitivity to chemotherapeutic drugs (**Figures 9A, B**). Based on the GDSC database, a comparative analysis of the half-maximal inhibitory concentration (IC50) values for six conventional chemotherapy agents—namely Linsitinib, Gefitinib, Dabrafenib, Cetuximab, BMS-754807, and XAV939—revealed consistently higher IC50 values in high-IGFL1 expression groups compared to low-expression groups (**Figures 9C–H**). This consistent elevation in IC50 across all compounds indicates a reduced therapeutic response among populations with elevated IGFL1 expression. Taken together, these findings suggest that higher IGFL1 expression is associated with diminished efficacy of chemotherapy in CRC.

### 3.9 Inhibition of IGFL1 inhibits CRC progression *in vitro*

**Figure 10:**
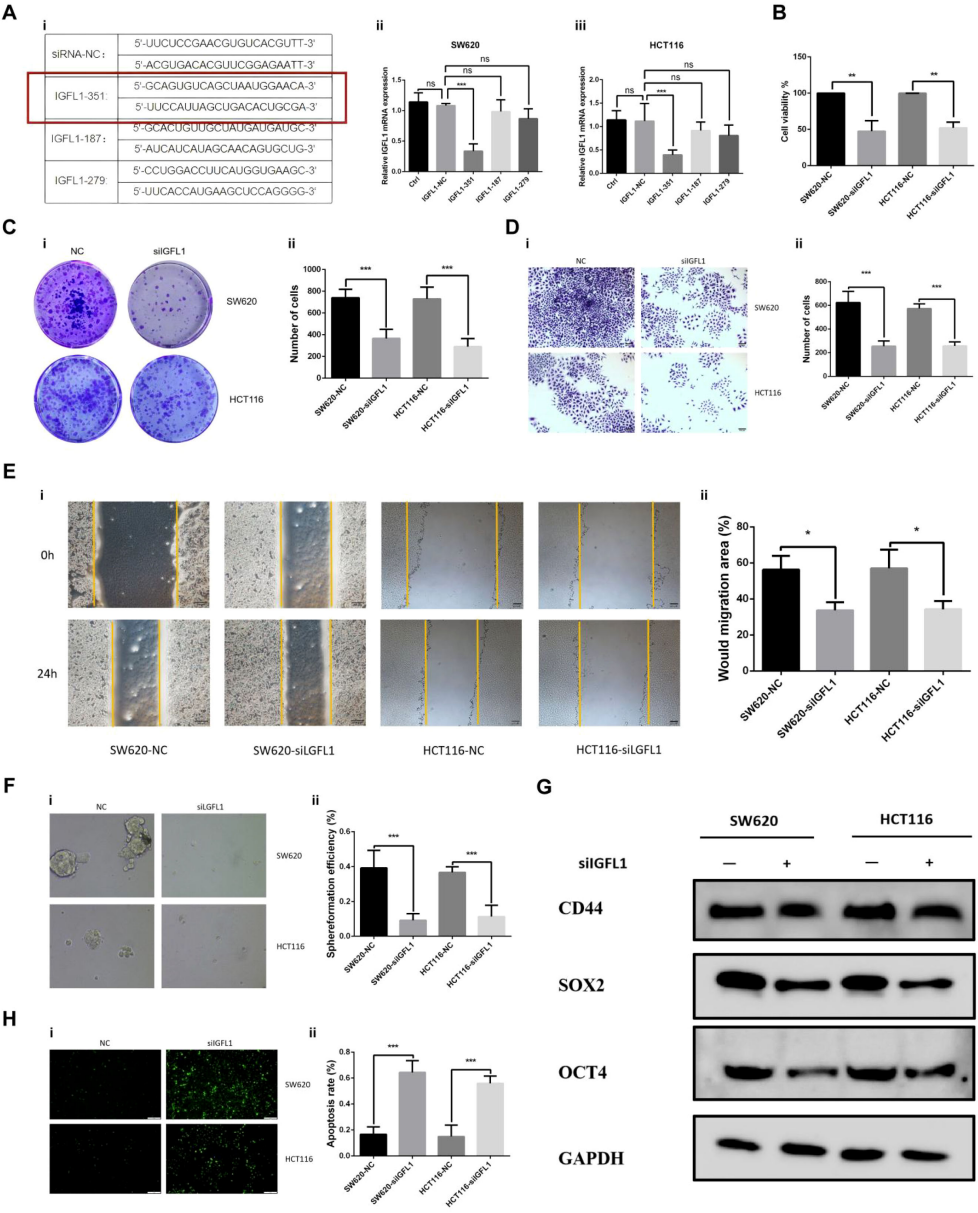
Suppression of IGFL1 impairs CRC malignant behaviors *in vitro*. **(A)** qRT-PCR assessment confirmed efficient knockdown of IGFL1 following plasmid transfection. Subsequent functional analyses revealed that IGFL1 downregulation significantly attenuated multiple oncogenic properties of CRC cells. Specifically, it suppressed cellular proliferation **(B)**, colony formation capacity **(C)**, invasive potential **(D)**, and migratory ability **(E)**. Furthermore, the self-renewal capability of cancer stem cells **(F)** and the expression of stemness-associated genes **(G)** were diminished. In contrast, apoptosis **(H)** was enhanced upon IGFL1 depletion.

Bioinformatics analyses identified IGFL1 as a potential key contributor to colorectal cancer pathogenesis, warranting further experimental investigation. Using two human colorectal carcinoma cell lines (SW620 and HCT116), we performed site-directed knockout targeting three distinct loci of the IGFL1 gene. RT-PCR analysis revealed a marked reduction in IGFL1 expression following knockout at locus 351 compared with control groups (**Figure 10A**). The stable cell lines generated from this construct were therefore designated as SW620-NC, SW620-silIGFL1, HCT116-NC, and HCT116-silIGFL1, and were used in subsequent functional assays.

Suppression of IGFL1 expression significantly impaired cellular proliferation (**Figure 10B**). Consistent with this, colony formation assays demonstrated a notable decrease in colony-forming capacity upon IGFL1 depletion (**Figure 10C**). Transwell invasion assays further indicated that IGFL1 knockdown substantially reduced the invasive potential of the cells (**Figure 10D**). In wound healing assays, IGFL1-silenced cells exhibited progressively diminished migratory ability compared with controls over the observation period (**Figure 10E**). Moreover, spheroid formation assays and western blot analysis revealed that IGFL1 ablation markedly attenuated the self-renewal capacity of colorectal cancer cells, accompanied by significant downregulation of stemness-associated markers (CD44, SOX2, and OCT4) (**Figures 10F, G**). Finally, apoptosis assays showed a pronounced increase in apoptotic rate following IGFL1 knockout (**Figure 10H**).

Collectively, these results indicate that IGFL1 suppression significantly impedes CRC progression *in vitro*, attenuating proliferative, invasive, migratory, and self-renewal capacities while promoting apoptosis.

### 3.10 IGFL1 knockdown inhibition lipophagy in CRC cells

**Figure 11:**
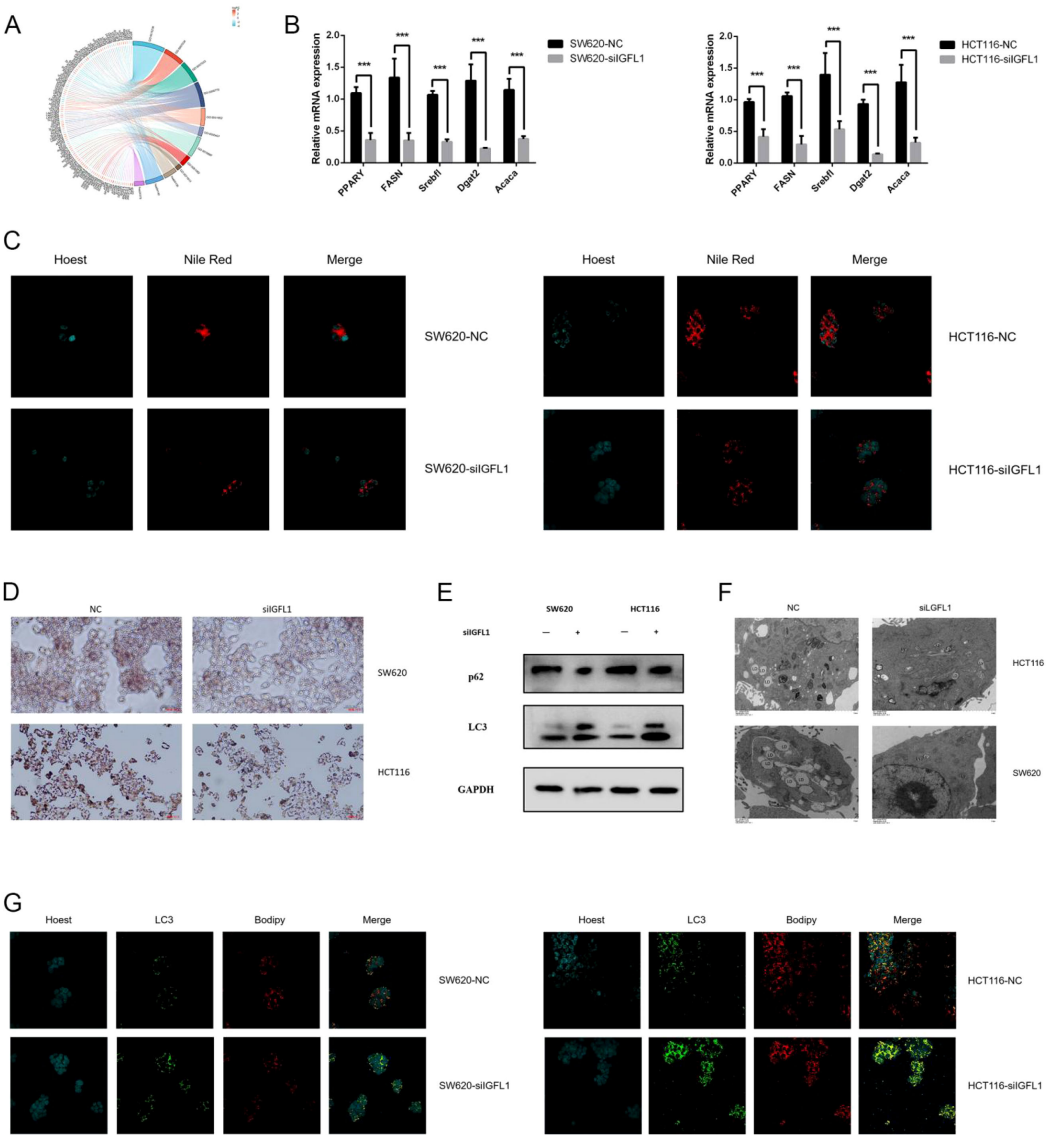
IGFL1 knockdown inhibition lipophagy in CRC cells. **(A)** GO and KEGG enrichment analyses of core differentially expressed genes in the transcriptome revealed associations between IGFL1 expression and cholesterol metabolism, autophagy pathways, and ATP hydrolysis activity. **(B)** RT-PCR demonstrated that IGFL1 knockout significantly downregulated the expression of cholesterol metabolism-associated genes. **(C, D)** Nile Red and Oil Red O staining assays indicated a marked reduction in intracellular lipid droplet content following IGFL1 knockout. **(E)** The expression of genes associated with the autophagy pathway was significantly upregulated by IGFL1 knockout, as revealed by Western blot analysis. **(F)** TEM demonstrated autolysosome proliferation alongside a significant decrease in lipid droplet quantity post-IGFL1 knockout. **(G)** Immunofluorescence analysis demonstrated enhanced co-localisation of LC3 with lipid droplets in IGFL1-knockout cells. *** means p < 0.001.

To elucidate the underlying mechanisms, we conducted mRNA sequencing using IGFL1-knockout cells alongside their corresponding wild-type controls. Comparative analysis identified 364 upregulated and 3121 downregulated genes between the two cell lines. Differentially expressed genes (DEGs) were defined based on a threshold of |log2FC| > 2 and an adjusted p-value < 0.01. Subsequent Gene Ontology (GO) and Kyoto Encyclopedia of Genes and Genomes (KEGG) pathway enrichment analyses revealed that the DEGs were predominantly enriched in cholesterol metabolism, autophagy-related pathways, and ATP hydrolysis activity (**Figure 11A**).

We then examined the expression of key genes involved in cholesterol metabolism and autophagy via RT-PCR and Western blotting. The results indicated that, compared with the control group, IGFL1 knockout led to a significant downregulation of lipid metabolism-related genes, while the expression of autophagy-related genes was markedly upregulated (**Figures 11B, E**). These findings were further supported by Nile Red and Oil Red O staining, which showed a pronounced reduction in intracellular lipid droplets following IGFL1 knockdown (**Figures 11C, D**).

To investigate whether IGFL1 influences colorectal carcinogenesis through the regulation of lipophagy, we performed transmission electron microscopy (TEM). The analysis revealed a substantial increase in autolysosomes accompanied by a decrease in lipid droplet abundance in IGFL1-knockdown cells (**Figure 11F**). In parallel, immunofluorescence staining demonstrated enhanced co-localisation of LC3, a canonical autophagosome marker, with lipid droplets upon IGFL1 knockdown (**Figure 11G**).

Collectively, these results suggest that IGFL1 may promote colorectal carcinogenesis by suppressing lipophagy.

## 4 Discussion

Previous studies have established that IGFL1 is closely associated with the pathogenesis and progression of multiple malignancies. In particular, it has been shown to promote tumour cell proliferation, support cell survival and differentiation, and inhibit apoptosis (12–14). However, its role in the development of colorectal cancer (CRC) remains poorly understood. Through integrated bioinformatic analysis and experimental validation, this study elucidates the function and potential regulatory mechanisms of IGFL1 in CRC.

Bioinformatic analysis indicated that IGFL1 expression is elevated in a wide range of cancers, with particularly marked upregulation observed in CRC. Diagnostic ROC analysis further identified IGFL1 as a promising biomarker for CRC detection. These findings were corroborated by IHC staining of paired tumour and adjacent non-tumour tissues from 50 patients with CRC, which demonstrated consistently higher expression of IGFL1 in malignant tissues compared with matched paracancerous samples, where expression was low or absent. Collectively, these results suggest that IGFL1 may contribute to CRC progression and underscore the importance of further investigation into its mechanistic role in colorectal carcinogenesis.

To validate the diagnostic significance of IGFL1 and explore its wider clinical relevance, we further examined its relationship with disease severity and patient outcomes. Using all available colorectal cancer datasets from the TCGA database, we assessed the association between IGFL1 expression and clinical progression. Our results revealed that higher IGFL1 expression correlates with more advanced clinical and pathological stages, as well as greater likelihood of lymph node metastasis, distant metastasis, and perineural invasion. This indicates that elevated IGFL1 expression is closely related to the pathogenesis, progression, metastasis, and invasive potential of CRC, consistent with our *in vitro* cellular experiments. Moreover, survival analysis showed that elevated IGFL1 expression in CRC patients is associated with a poorer prognosis and may function as an independent prognostic factor influencing PFI in these patients. Together, these findings support the potential use of IGFL1 as a clinicopathological indicator for identifying patients with unfavourable prognostic outcomes. Additionally, the nomogram we developed provides a novel and practical prognostic tool to aid clinicians in predicting 1-, 3-, and 5-year survival rates in CRC patients.

We subsequently utilised a state-of-the-art algorithm to analyse the role of IGFL1 in the immune microenvironment of CRC, leveraging all accessible CRC datasets from TCGA. This analysis specifically examined the correlation between IGFL1 expression and both immune cell infiltration and immunoscore. To validate the TCGA-based observations, an external single-cell RNA sequencing dataset of CRC from the TISCH database was also incorporated. Our comprehensive assessment revealed that IGFL1 expression in CRC is significantly associated with macrophages, CD4^+^ T cells, dendritic cells, natural killer cells, and B cells, underscoring the complexity of the IGFL1-modulated immune landscape in CRC pathogenesis. It is well established that IGFLR1, a high-affinity receptor for IGFL1, plays a significant role in the colorectal cancer immune microenvironment. IGFLR1 has been identified as a novel prognostic biomarker in CRC, exhibiting differential expression in tumour tissues and a significant association with improved patient outcomes and immune cell infiltration. Further analyses indicate that IGFLR1 serves as an independent prognostic factor, contributes to an effective prognostic stratification model, and demonstrates notable co-expression with key immune-related pathways such as the interferon response (INFR) (27). In a related context, a study published in Cell by Christian M. Schürch et al. employed multiplexed imaging to spatially profile the immune tumour microenvironment (iTME) in colorectal cancer. The authors identified distinct cellular neighbourhoods and reported that specific enrichment of PD-1^+^CD4^+^ T cells within a granulocyte-associated neighbourhood was correlated with improved survival. Their analysis further revealed that poorer patient outcomes were associated with fragmentation of critical immune cellular neighbourhoods and disrupted spatial coordination across the iTME (28). Additionally, previous research has shown that acidosis in colorectal cancer cells can suppress the activity of anti-tumour immune cells and impair the phagocytic function of tumour-associated macrophages (TAMs), while nutrient deprivation promotes the differentiation of regulatory T cells (Tregs) and M2-like macrophages (29). Collectively, these findings highlight the influential role of the immune microenvironment in CRC progression, which will constitute a major focus of our subsequent investigations.

In recent years, immunotherapy has become a prominent focus in the management of CRC, encompassing modalities such as immune checkpoint blockade (ICB), adoptive cell therapy (ACT), cancer vaccines, and cytokine-based treatments. These approaches seek to enhance the immune system’s ability to recognise, target, and eliminate malignant cells. It has been proposed that combining radiotherapy, chemotherapy, and various immunotherapeutic strategies could substantially improve the immune-mediated eradication of colorectal tumour cells. Furthermore, in light of the immunologically “cold” nature of certain CRC subtypes, key strategies to enhance immunotherapy efficacy in these malignancies have been proposed (30). Against this background, we analysed the correlation between IGFL1 expression levels (high vs. low) and both TIDE scores and ICB responsiveness. Previous studies have reported upregulated expression of CTLA4, BTLA, TIM-3, and LAG-3 in CRC patients, which correlates with cancer stage and survival outcomes, underscoring the diagnostic and prognostic relevance of immune checkpoint molecules in colorectal cancer (31). Hansong Lee et al. identified the LGALS9–HAVCR2 axis as a key pathway in CRC, with HAVCR2+ NK cells exhibiting suppressed cytotoxic function, indicative of impaired immune surveillance (32). Collectively, this study identifies high IGFL1 expression as a potential predictive biomarker for suboptimal immunotherapy response in CRC. Future research should focus on validating these mechanisms in preclinical models and exploring combinatorial treatment strategies to overcome immunotherapy resistance in IGFL1-high CRC subtypes.

Literature suggests that the association between differential gene expression and reduced response to immunotherapy may be linked to its co-occurrence with specific genetic alterations (33). These underlying genomic features may collectively foster a tumour microenvironment that is both immunosuppressive and permissive for oncogenic progression. Accordingly, we analysed the genetic alteration profile of IGFL1 in CRC. Our analysis demonstrated that missense mutations represent the most frequent alteration in IGFL1 in CRC patients. Concurrent with elevated IGFL1 expression, we observed an increased mutation burden in APC, TP53, TTN, and KRAS. Mutation and inactivation of APC constitute a critical early event in colorectal tumourigenesis. Truncating alterations in APC lead to constitutive activation of the Wnt signalling pathway and disruption of diverse cellular processes, thereby promoting colorectal cancer development (34). Furthermore, TTN mutations have been recognised as risk factors for colorectal cancer, influencing the tumour microenvironment, tumour stemness, and conferring adverse prognosis (35). Collectively, this evidence may inform future strategies for multi-gene targeted combination therapies.

Given the role of IGFL1-associated genetic alterations in shaping tumour biology, we further investigated their potential influence on therapeutic efficacy. Accordingly, we evaluated whether IGFL1 expression affects responses to conventional pharmacological agents. Drug sensitivity analysis demonstrated that elevated IGFL1 expression enhances resistance to several chemotherapeutic agents, including linsitinib, gefitinib, dabrafenib, cetuximab, BMS-754807, and XAV939. As these drugs are commonly used in CRC therapy, patients with high IGFL1 expression may benefit from avoiding the aforementioned treatments (36). Previous studies have shown that TSPYL2 reduces gefitinib resistance and impairs DNA repair by inhibiting SIRT1-mediated deacetylation of FOXO3 (37). Furthermore, Jue Wang et al. developed a hydrogel-based system to selectively deplete copper in BRAF<V600E>-mutant colorectal cancer cells, effectively overcoming resistance to dabrafenib/cetuximab and improving therapeutic outcomes (38). These findings underscore the importance of considering IGFL1 status when selecting clinical chemotherapy regimens for CRC patients with alterations in the IGFL1 gene.

To validate the findings from the bioinformatic analysis, we used two CRC cell lines, SW620 and HCT116, in which IGFL1 expression was knocked down by stable transfection with shRNA. The results showed that IGFL1 knockdown decreased cell viability, proliferation, migration, invasion, and clonogenic ability, while increasing apoptosis. Additionally, the expression of stemness-related genes was reduced. Taken together, these results indicate that IGFL1 promotes CRC tumorigenesis and progression, and enhances the invasive and metastatic capacity of CRC cells.

To further elucidate the potential mechanisms by which IGFL1 influences the pathogenesis and progression of colorectal cancer (CRC), transcriptome sequencing was performed. The analysis showed that differentially expressed genes were predominantly enriched in cholesterol metabolism, autophagy pathways, and ATP hydrolysis activity. It has been reported that B7H3 promotes ferroptosis resistance via modulation of SREBP2-mediated cholesterol metabolism, thereby contributing to colorectal carcinogenesis (39). In addition, studies indicate that YAP suppresses autophagy through upregulation of Bcl-2 expression, which in turn facilitates CRC progression (40). Furthermore, Mengchen Shi et al. demonstrated that ATPase and AMPase activities are upregulated in CRC, with significant diagnostic relevance. Combined detection of CEA, ATPase, and AMPase may thus offer a novel strategy for CRC screening (41). Collectively, these enriched pathways provide important mechanistic clues linking IGFL1 to colorectal cancer development and progression.

Research was conducted to elucidate the underlying mechanisms. Lipophagy, a selective form of autophagy, has emerged as a novel lipid metabolism pathway that has attracted considerable interest in recent years (42). It plays a key role in regulating intracellular lipid storage, modulating free intracellular lipid levels, and maintaining energy homeostasis (43). However, under different physiological or pathological conditions, lipophagy exhibits divergent functions. Some studies have shown that lipophagy can inhibit disease onset (44–46), whereas others indicate that it may promote disease progression or contribute to drug resistance (47–49). Furthermore, Haimeng Yin et al. (50) demonstrated that lipophagy is involved in tumour metastasis. These findings underscore the complexity of lipophagy in physiological and pathological contexts, highlighting the need for further investigation.

In this study, we demonstrate that IGFL1 promotes the occurrence and progression of colorectal cancer by suppressing lipophagy; however, the precise molecular mechanisms remain to be fully elucidated. We propose two potential mechanistic hypotheses: (I) IGFL1 may act in an autocrine or paracrine manner by binding to cell surface receptors, leading to activation of the PI3K/Akt/mTORC1 signalling pathway. This cascade subsequently phosphorylates and inhibits the ULK1 complex—a critical initiator of autophagy—thereby impeding lipophagy (51, 52). Alternatively, (II) intracellularly retained IGFL1 may directly or indirectly interact with lipid droplet-associated proteins (e.g., PLIN2), autophagy adaptors (e.g., p62/SQSTM1), or the AMPK complex, thereby disrupting the formation of lipophagosomes. Impaired lipophagy results in abnormal intracellular lipid droplet accumulation. These accumulated lipids may be converted into pro-inflammatory mediators or signalling lipids (such as phosphatidic acid), or may induce oxidative stress, ultimately driving cancer cell proliferation, invasion, and metastasis (53–56). The observed cytoplasmic localisation of IGFL1 provides a spatial basis for mechanism (II), or alternatively, for the intracellular assembly of downstream signalling complexes following receptor activation.

While our study incorporated comprehensive bioinformatics analyses and *in vitro* experimental validation regarding the role of IGFL1 in CRC, several limitations should be acknowledged. First, the exploration of IGFL1 within the CRC immune microenvironment, its predictive value for immunotherapy, and its association with drug sensitivity remain at the bioinformatics level. Although our findings indicate correlations between IGFL1 expression and immunotherapy-related biomarkers as well as drug response profiles, these observations lack experimental confirmation. Second, although we confirmed the oncogenic function of IGFL1 in CRC, the relatively limited sample size may have introduced selection bias. Third, while this study provides preliminary evidence that IGFL1 influences CRC pathogenesis via lipophagy, the precise underlying molecular mechanisms remain unclear. Moreover, experimental validation of lipophagy was restricted to cellular models and has not yet been extended to *in vivo* systems. Additionally, due to the currently unknown regulatory pathways involved, rescue experiments or pathway-specific interventions were not performed, which somewhat limits the robustness of our conclusions.

In future work, we plan to first investigate the specific mechanism by which IGFL1 regulates lipophagy based on our proposed hypothesis, followed by mechanistic validation accordingly. Second, we will expand the sample size and endeavour to establish a multicentre collaborative clinical cohort. Stratifying patients according to IGFL1 expression levels will allow more in-depth analysis of the immune microenvironment and therapy resistance, potentially improving immunotherapeutic strategies. Finally, we will further examine the role of lipophagy in drug resistance, with the long-term goal of modulating lipophagic activity to overcome chemoresistance, enhance drug sensitivity, and improve patient outcomes during conventional chemotherapy.

## 5 Conclusions

This study demonstrates that IGFL1 is upregulated in CRC and correlates with adverse clinicopathological features and poorer prognosis. IGFL1 promotes oncogenic functions in CRC by modulating tumour immunity, drug sensitivity, and key cellular behaviours including proliferation, migration, invasion, clonogenicity, and epithelial–mesenchymal transition (EMT). *In vitro* experiments confirm that knockdown of IGFL1 inhibits these malignant processes. Notably, IGFL1 appears to facilitate CRC progression through the suppression of lipophagy. These findings highlight IGFL1 as a potential diagnostic and prognostic biomarker in CRC. Furthermore, IGFL1 may represent a viable therapeutic target, providing a practical avenue for personalised CRC treatment.

## Data Availability

The datasets presented in this study can be found in online repositories. The names of the repository/repositories and accession number(s) can be found in the article.
